# Supplementary material for: Effect of C-reactive protein and serum amyloid A point-of-care testing on antibiotic prescribing for acute respiratory-tract infections at village clinics in China: A study protocol for a cluster randomised controlled trial
Source: PLoS One. 2025 Sep 8;20(9):e0331646. doi: 10.1371/journal.pone.0331646 (PMC12416646; doi:10.1371/journal.pone.0331646)
Supplement: S2 File — (PDF) [file pone.0331646.s002.pdf]

Title of study

Intervention strategy and empirical evaluation of rational use of antibiotics for patients with acute respiratory tract infection in village clinic based on point-of-care testing

Research setting

Department of Social Medicine and Health Management, School of Public Health, Huazhong University of Science and Technology

Principal investigator

Principal investigator’s name and address

Background

Antimicrobial resistance is a globally recognised public health threat, and one of its primary drivers is the significant increase in antibiotic prescriptions. In China, the use of antibiotics is common in rural areas. Acute respiratory infections (ARIs), which include symptoms such as coughing and fever, are among the primary reasons for antibiotic prescriptions in village clinics. However, only a small proportion of patients with bacterial ARIs actually require antibiotic treatment. Identifying effective interventions to reduce the inappropriate use of antibiotics for ARIs in village clinics could significantly improve antibiotic use in China. In recent years, certain biomarkers, such as C-reactive protein (CRP), have increasingly been utilised to inform antibiotic prescribing practices for ARIs. Although CRP POCT is a valuable tool in diagnosing bacterial infections, its specificity is limited, as viral infections can also lead to elevated CRP levels. Additionally, since CRP is part of a dynamic and continuous inflammatory process, a single CRP measurement, especially at low concentrations, may erroneously classify an infection as viral rather than bacterial, potentially delaying appropriate antibiotic treatment. The clinical significance of serum amyloid A (SAA) is promising as changes in its levels play a crucial role in the early diagnosis of infectious diseases. SAA demonstrates high diagnostic accuracy for viral infections and is significantly elevated during the acute phase of such infections. By assessing the degree of SAA and combining it with CRP, it is possible to differentiate between bacterial and viral infections more accurately. This indication of a combined biomarker addresses the limitations of currently used single biomarkers, which often fail to indicate viral infections specifically. Despite its significant potential, the evidence supporting the use of CRP&SAA POCT for antibiotic management of ARIs in primary care settings remains insufficient. The simplicity, rapidity, low cost, and high accuracy of CRP&SAA POCT make it particularly suitable for remote primary healthcare settings, such as village clinics in China.

Methods

Design

This is a pragmatic, parallel-group, cluster randomised controlled trial designed to evaluate the impact of a CRP&SAA POCT-based comprehensive intervention on

antibiotic prescribing for patients with ARIs in Chinese village clinics. This study will be conducted over a period of six months across 40 village clinics, with randomization stratified by township. CRP&SAA POCT will be implemented in 20 village clinics within the intervention arm. This will include additional training for village doctors on the operations of CRP&SAA POCT, which encompasses centralised training, the distribution of training manuals, and desk reminders. The aim is to help village doctors understand the significance of CRP&SAA POCT in guiding antibiotic prescriptions and to familiarise them with the specific operational procedures. Additionally, patient education materials will be provided to assist patients in understanding how CRP&SAA POCT can aid in their diagnosis and treatment. The control arm will not receive any intervention and will continue with usual care.

Aims and hypotheses

In this study, we aim to evaluate the effectiveness of a comprehensive intervention based on CRP&SAA POCT, which we hypothesised would reduce the inappropriate use of antibiotics for ARIs in Chinese village clinics.

Setting

As the primary providers of health care services in rural China, village clinics address various aspects, including basic medical services, preventive health care, public health services, and drug supply, with the goal of meeting the fundamental medical and public health needs of rural residents. This study will be conducted in 40 village clinics across 15 townships and 4 sub-district offices (equivalent to townships) in Xiantao, Hubei Province. Xiantao is situated in central China's Hubei Province, with a population of approximately 1.11 million permanent residents. The city administers 15 townships and 4 sub-district offices. Each village clinic employs 1 to 2 upgraded community health workers, commonly referred to as 'village doctors'. All village doctors have successfully passed the national doctor qualification examination and have obtained either a certificate of practicing physician qualification or a certificate of practicing assistant physician qualification. This includes qualifications for practicing as a physician or assistant physician in the clinical category, as well as the rural general practice assistant physician qualification.

Intervention

According to national and international guidelines, the self-limiting virus ARIs should not be treated with antibiotics. The intervention aimed to guide the prescribing behavior of ARIs by doctors in Chinese village clinics through the use of CRP&SAA POCT. The purpose and design of this study adhered to policy requirements for antibiotic prescribing and are subject to routine oversight by local health authorities. To facilitate the implementation of CRP&SAA POCT, the intervention design is guided by the Theoretical Domain Framework (TDF), an emerging approach developed from a diverse array of theories related to behavior change. The TDF comprises 14 theoretical domains and is widely utilised in implementation studies to

design interventions and examine factors that influence their effectiveness. Several theoretical areas within the TDF were identified as crucial for CRP&SAA POCT-guided antibiotic prescribing, where the content of relevant behavior change techniques and interventions was specifically targeted.

CRP&SAA POCT

Serum Amyloid A/C-Reactive Protein Combined Assay Kit (Immunochromatography) will be supplied to all village clinics in the intervention arm for the target population (ARIs patients). Since the purpose of this study is to determine whether antibiotic prescribing for ARIs can be guided by the use of CRP&SAA POCT, the intervention arm should be encouraged to utilise CRP&SAA POCT for all target populations (patients with ARIs). This recommendation is suggested but not mandated. The control arm will not receive any intervention (usual care). Village doctors will decide whether to prescribe antibiotics to patients with ARIs based on the recommended threshold for CRP&SAA levels (mg/L). As there is currently no specific cut-off threshold for CRP and SAA, our recommended threshold was developed in conjunction with previous studies and expert consensus in China to align more closely with clinical practice. In order to further encourage village doctors to utilise CRP&SAA POCT, we simulated real-world conditions. According to the reimbursement policy, the Rural Cooperative Medical Care in China, provides a 60% reimbursement for visits to village clinics. Each patient with an ARI utilizing the CPR&SAA POCT, originally priced at 14 yuan (approximately US $1.92), will ultimately pay 5 yuan (approximately US $0.68) to the village clinic.

Healthcare worker training

After randomization, we will implement a unified and centralised training program for doctors in all village clinics within the intervention arm. The training will cover the following topics: (1) the operation process for CRP&SAA POCT; (2) recommended thresholds for CRP&SAA, along with practical case applications; and (3) clinical evaluation and management of ARIs. Following the intensive training, the research team will provide on-site guidance for the use of CRP&SAA POCT in each village clinic within the intervention arm for one week. This support will ensure that each village doctor is capable of independently operating the CRP&SAA POCT. Training will be conducted by the research team, and an online communication group will be established to address any questions that village doctors may have during the follow-up process. The research team will distribute a CRP&SAA POCT-based village clinic guidance manual on the rational use of antibiotics for ARIs to each village clinic in the intervention arm. This guideline includes an introduction to ARIs, standardised procedures for their diagnosis and treatment, CRP&SAA POCT-based clinical diagnosis and treatment pathways for ARIs, a brief overview of the principles of CRP&SAA POCT, and illustrated operational steps. Desktop reminders featuring the recommended thresholds from CRP&SAA will be distributed to the intervention arm. Village doctors in the intervention arm will be informed that the CRP&SAA recommended threshold serves only as a suggestion, and that antibiotic prescribing

must ultimately be determined based on the clinical judgment. If a village doctor believes that patients with ARIs require referral to a higher level of care, the referral should be done immediately, and CRP&SAA POCT should not be utilised. At the beginning of each month following the intervention, the research team will gather prescription information for ARIs from all village clinics within the intervention arm. The team will conduct a comprehensive assessment to determine whether village doctors are prescribing antibiotics in accordance with the CRP&SAA POCT threshold. Subsequently, the team will provide feedback to the relevant village clinics and administrative staff.

Patient education

For the village clinics in the intervention arm, patients presenting with ARIs will receive a popular science leaflet detailing the role of CRP&SAA POCT in promoting the rational use of antibiotics for ARIs, as well as the risks associated with antibiotic overuse. According to the information provided in the publicity leaflet and the advice of the village doctor, patients with ARIs (or their legally appointed agents) will determine whether to consent to CRP&SAA POCT based on their individual circumstances.

Usual care

Village clinics in the control arm will continue to prescribe antibiotics to patients with ARIs in accordance with the current national guidelines and usual care in village clinics. In usual care, village doctor will diagnose and treatment of ARIs based on their clinical experience and existing knowledge, and make decision on whether to prescribe antibiotics. Notably, the village doctors in the control arm will not receive any education on ARIs and the appropriate use of antibiotics, and will not receive the Serum Amyloid A/C-Reactive Protein Combined Assay Kit.

Allocation and blinding

The 40 village clinics will be randomly assigned to one of the two arms, stratified by township, to prevent any imbalance in distribution among townships, while considering potentially significant variation in outcomes between townships. Randomisation will be conducted using R version 4.0.5 (R Foundation for Statistical Computing). ARIs patients and village doctors will not be blinded to the treatment; however, measures will be implemented to ensure a blinded outcome evaluation by employing the 'PROBE' design.

Eligibility

Eligibility criteria for clusters

Village clinics with an annual outpatient volume exceeding 2000, an average of 10 or more patients per week presenting with ARIs, and licensed prescribers are considered eligible for selection for the intervention. Annual outpatient prescriptions and average weekly visits for ARIs will be verified by obtaining prescription data from all village

clinics for the previous year, as documented in the information section of the local health board.

Eligibility criteria for participants
The target population of this study included (1) patients of all ages diagnosed by a village doctor with ARIs (including upper and lower respiratory infections); and (2) patients who present with ≥1 acute respiratory symptoms (including cough, rhinitis (sneezing, nasal congestion or runny nose), sore throat, shortness of breath, wheezing or abnormal auscultation). Patients with non-respiratory diseases or those with severe clinical symptoms requiring referral to a higher-level institution are excluded from the target population.

Outcomes
Primary outcome
The primary outcome is the proportion of patients who are diagnosed with ARIs and prescribed antibiotics during their initial visit (defined as no prescription record at the current institution within the preceding 14 days) in both study arms. This outcome serves as the primary indicator, reflecting the overall impact of a comprehensive intervention based on CRP&SAA POCT in guiding antibiotic use for patients with ARIs. Since most self-limiting ARIs are caused by viral infections that do not require antibiotic treatment, the decline in antibiotic prescribing rates suggests that village doctors are prescribing antibiotics more judiciously. The selection of this outcome is both feasible and reliable within the context of village clinics in China. This is due to the transition of prescriptions from traditional paper documents to electronic storage, which allows for the proper preservation of prescription records, thereby ensuring data integrity and traceability.

Secondary outcomes
Secondary outcomes will be extracted from the prescribing information related to the primary outcome, as well as from subsequent visit information. In particular, we included the proportion of participants using any form of Traditional Chinese Medicines as a secondary outcome. In our previous trials, we observed an increase in the use of Traditional Chinese Medicines, possibly as an alternative to antibiotics. These outcomes will include the following types:
1. The proportion of multiple antibiotic prescriptions in the intervention and control arms (specifically, the proportion of ARI prescriptions that include two or more antibiotics).
2. The intravenously injected antibiotic prescription rate (the proportion that contain any antibiotics delivered by intravenous injection).
3. The proportion containing any Traditional Chinese Medicines.
4. The mean cost of an ARI prescription, based on the cost of any medicines.
5. The mean cost of a consultation, based on all costs including medicines, tests and the consultation.

Patient safety indicator
We will evaluate whether the intervention appears to increase the incidence of adverse events. This may occur if antibiotics are more frequently withheld for appropriate conditions as a result of the intervention. To address this concern, we will use the proportion of patients with ARIs in both the intervention and control arms who are hospitalised in Xiantao City for ARIs or sepsis within 30 days following their initial ARIs visit, excluding those who are referred by village doctors during their initial consultations, as a safety indicator.

Sample size
Based on our exploratory study and previous research, the antibiotic prescription rate in rural primary healthcare institutions in China ranges from 50% to 80%. The minimum value of 50% is utilised as a conservative estimate of the conventional antibiotic prescription rate in the control arms. We conservatively estimate that CRP&SAA POCT intervention will lead to at least a 25% relative reduction in the antibiotic prescription rate in village clinics. Thus, to detect a reduction of 25% or more in the antibiotic prescription rate - specifically, an absolute reduction to 37.5% antibiotic prescription rate or lower - with 90% power, utilizing two-sided testing at the 5% significant level, and assuming a harmonic mean cluster size of 500 along with an intracluster correlation coefficient of 0.05, we estimate that we will require 17 village clinics per arm. Allowing for a 15% loss of prescription data and considering that 20 village clinics are required per arm, it was decided to include a total of 40 village clinics.

Internal pilot process
Before initiating the main trial, we will evaluate the feasibility of implementing the intervention through an internal pilot process. During the preparation period, 8 village clinics (from 4 townships) will be selected as pilot sites and randomly assigned to either the intervention or control arm. They will be followed up for one month to evaluate the feasibility of the following objectives: (1) whether all village doctors in the intervention arm can independently operate the CRP&SAA POCT; and (2) whether the doctors can effectively guide the antibiotic prescription for patients with ARIs based on the varying cut-off thresholds of the CRP&SAA POCT. We will adjust the implementation plan as necessary based on the insights gained from the internal pilot process.

Data analysis plan
All analyses will be conducted on the intention-to-treat (ITT) population, defined as all outpatient prescriptions issued by village clinics for ARI patients, regardless of the compliance of village doctors and patients with the intervention. For the primary and secondary outcomes, which are binary variables, we will employ Generalised estimating equations (GEEs) to estimate both relative and absolute measures of effect. The same methods will be used to analyse the mean cost of an ARI prescription and a consultation. The primary outcome will also be analysed in subgroups based on ARI

patient's age (1-15 / 16-65 / over 65 years old), ARI patient's sex (male / female), type of ARI (upper respiratory tract infection / lower respiratory tract infection), and season. Likelihood ratio tests will be employed to assess the heterogeneity of antibiotic prescribing across these different subgroups. We also will conduct per-protocol analyses, which included only ARI patients who received CRP&SAA POCT in village clinics within the intervention arm. Furthermore, modified poisson regression and linear regression models, which account for village-level clustering using the clustered sandwich estimator, will be employed to replicate all results and assess their robustness. No interim analyses are planned, and all outcomes will be analysed following data collection.

Process evaluation

A process evaluation will be conducted for all village clinics in the intervention arm. Our specific objectives for the process evaluation are: (1) to describe the health system and service delivery context in which the intervention was implemented; (2) to assess intervention fidelity; (3) to identify the barriers and facilitators in the implementation process and determine which mechanisms influence behavior change outcomes. The process evaluation of this study may serve as a foundation for planning similar projects in other low- and middle-income countries in the future. Our analysis will be based on the TDF while also allowing for the generalization of important newly discovered topics. The steps of our analysis include familiarizing ourselves with the text, reaching a consensus on the coding framework, indexing the content according to the framework, generalizing the findings, and interpreting the results. Nvivo Version 10 (QSR International Pty Ltd) will be utilised to manage the data. The quality of reporting in the qualitative study will be ensured by adhering to the Consolidated Criteria for Reporting Qualitative Research (COREQ).

Trial management

Prof. Xiaoxv Yin from the Huazhong University of Science and Technology and Prof. Xiaolin Wei from the University of Toronto will be the co-guarantees of the trial and will have full access to the trial dataset. We have established an independent research team composed of the project leader, representatives from the information section of the local health board, and members involved in the specific implementation project. This team will design and monitor the trial, review its progress, and determine any necessary changes to the protocol.

Dissemination

This initiative is supported by funding from the University of Toronto-Huazhong University of Science and Technology Joint Seed Fund. Throughout the research project, we will implement a CRP&SAA POCT-based comprehensive intervention in village clinics across China to improve antibiotic use in ARIs. We will produce policy briefs and recommendations to influence current practices in primary healthcare institutions. Additionally, the findings will be published in internationally peer-reviewed journals through peer-reviewed publications and presented at academic

conferences in China, Canada, and other countries. The total lifespan of the study will be 24 months.
